# Epigallocatechin‐3‐O‐gallate modulates the diversity of gut microbiota in ovariectomized rats

**DOI:** 10.1002/fsn3.1419

**Published:** 2020-01-27

**Authors:** Beilin Zhang, Jinpeng Wang, Qiyan Wei, Yi Liu, Huiwen Zhang, Xiaohui Chen, Kun Xu

**Affiliations:** ^1^ Department of Physiology College of Basic Medical Sciences Jilin University Jilin China; ^2^ Department of Cardiology the Second Hospital of Jilin University Jilin China; ^3^ College of Chinese Medicinal Materials Jilin Agricultural University Jilin China; ^4^ Department of Nutrition and Food Hygiene School of Public Health Jilin University Jilin China

**Keywords:** antianxiety effect, EGCG, intestinal microbiota, ovariectomized rat

## Abstract

Epigallocatechin‐3‐O‐gallate (EGCG) exists as one of the major active components of green tea and has been studied extensively; however, the relationship between EGCG and the changes in the gut microflora of ovariectomized (OVX) rats as a model of menopause women have not yet been studied. Female Wistar rats were fed on a maintenance material diet and underwent either ovariectomy or SHAM surgery. The ovariectomized rats were divided into OVX group with the treatment of placebo or EGCG group which was treated with EGCG by oral gavage. After 8 weeks of treatment, anxiety‐like behaviors were assessed using elevated plus maze test (EMP) and open field test (OFT). The serum estradiol concentration was assayed through ELISA. High‐throughput V3–V4 16S rDNA sequencing was conducted to assess the microbial diversity in fecal samples collected from all rats. EGCG, at a concentration of 10 mg/kg, caused behavioral changes in rats similar to anxiety. In EPM, OVX rats spent less time in open arms than SHAM group rats and EGCG group rats (*F* = 16.043, *p* < .01). In OFT, the total travelled distance and the number of entries for EGCG group were higher compared with OVX group (*F* = 30.939, H = 13.107, respectively; *p* < .01). In addition, the distribution and composition of intestinal microflora in rats changed after ovariectomy. EGCG modulated the diversity of gut microbiota in OVX group at the phylum and the genus levels. Our results suggested that the composition of gut microbiota and anxiety in OVX rats were simultaneously affected by EGCG, and therefore, the two conditions might be strongly related, yet the deeper mechanistic links need further exploration.

## Graphical abstract

EGCG exhibits antioxidative, anti‐inflammatory, and anticarcinogenic properties and other biological activities. It is expected that EGCG could have a great potential in ameliorating the symptoms of anxiety in postmenopausal women.

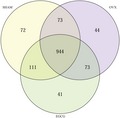

## 1 INTRODUCTION

Menopause is an important life transition that all women go through, and it accompanies many significant physical and psychological changes, such as bone loss (Boschitsch, Durchschlag, & Dimai, 2017), obesity (Goncalves, Silveira, Campos, & Costa, 2016), depression, and anxiety (Bromberger & Epperson, 2018; Gracia & Freeman, 2018). The reason why menopausal women undergo such changes is directly related to the low level of endogenous estrogens. Furthermore, 65% of postmenopausal women have metabolic dysfunction, which is attributed to the association of the gut microbiota with lack of estrogen (Leeners, Geary, Tobler, & Asarian, 2017). It is reported that the gut microbiota modulates estrogen homeostasis via enterohepatic circulation (Flores et al., 2012). Thus, the effect of menopause on gut microbiota has attracted a lot of attention.

Accumulating evidence from previous studies suggests that the gut microbiota regulates mood disorders, including the two most prominent neuropsychiatric disorders that affect millions of people worldwide: anxiety and depression, via a microbiota–gut–brain axis (Rieder, Wisniewski, Alderman, & Campbell, 2017). Different laboratories and studies have reported that germ‐free (GF) mice exhibit consistently reduced anxiety symptoms (Clarke et al., 2013; Neufeld, Kang, Bienenstock, & Foster, 2011). It was also displayed by another study that the anxiety‐like and depression‐like behaviors were reduced in healthy male Balb/C mice after feeding *L. rhamnosus* (Bravo et al., 2011), which resulted in altered gut microbiota. Furthermore, the related human study also supports the relationship between microbiota and anxiety (Stevens et al., 2018).

Estrogen replacement therapy was tested as the predominant means to induce antianxiety effects in several studies (Hiroi & Neumaier, 2011; Weiser, Foradori, & Handa, 2008). However, its side effects are inevitable, including increased thrombosis risk, inducement of breast cancer, and serious mental disorder (Rozenberg, Vandromme, & Antoine, 2013). It is very necessary to develop alternative approaches that are more effective and safer. In recent years, the search for effective natural antidepressant and antianxiety agents from plants has gained much interest in the field of psychotropic drug research and development. Tea contains a lot of polyphenols, which exhibit strong antioxidant capacities. The main component of tea polyphenols is catechin. Around 50%‐60% catechins found in tea are (‐)‐Epigallocatechin‐3‐gallate (EGCG). EGCG exhibits antioxidative, anti‐inflammatory, and anticarcinogenic properties and other biological activities (Higdon & Frei, 2003; Pan, Lin‐Shiau, Ho, Lin, & Lin, 2000). Furthermore, it has been reported that EGCG could improve bone microarchitecture and reduce stress of oxidation in bladder for ovariectomized (OVX) rats, whose bladder cells were excessively active due to surgical menopause (Chen et al., 2013; Juan et al., 2012). Park et al. (2010) suggested that EGCG could reverse caffeine‐induced anxiogenic‐like effects in rats. Therefore, EGCG may exhibit antianxiety effects in ovariectomized rats. The changes caused by estrogen depletion in OVX rodents have many characteristics similar to changes in menopausal women, such as weight gain, adipose tissue inflammation, anxiety, depression, and hormone changes. Therefore, OVX rodents may be a suitable model for studying postmenopause. However, how EGCG influences microbiome environments in the gastrointestinal tract of OVX rats is still unrevealed. The current study was planned for verifying the effects of EGCG on the diversity of gut microbiota in addition to anxiety in OVX rats.

## 2 MATERIALS AND METHODS

### 2.1 Animal preparation

All animal usage in this study was approved and supervised by the Institutional Animal Care and Use Committee of Jilin University. Female Wistar rats (250 ± 20.0 g, 10 weeks) were acquired from the Changchun Yi Si Laboratory Animal Technology Co., Ltd. (SCXK‐(JI) 2016–003). All rats were fed with standard lab chow in a constant light/dark cycle (12h/12h) at room temperature and 70% relative humidity during the course of the experiments. This study was subject to the Institutional Guidelines for Animal Research; and the authors obeyed the Guide for the Care and Use of Laboratory Animals published by the US National Institutes of Health (NIH Publication No. 85–23, revised 1996). A total of 30 rats were randomly selected and sorted into three groups: the group of SHAM surgery (SHAM group, *n* = 10), bilateral OVX group (*n* = 10), and EGCG‐treated group (EGCG group, *n* = 10). Individual of EGCG group was treated with EGCG (Shanghai Yuan Ye Biotechnology Co., Ltd.; dissolved in saline) at 10 mg/kg through oral gavage once a day. SHAM and OVX individuals were treated with the same amount of saline by the same means of administration. The ovaries of OVX and EGCG rats were surgically removed, and the procedures including cutting two minimal incisions on the back of each rat under 10% chloral hydrate (0.35 ml/kg) induced anesthesia. The SHAM surgery was carried out by exposing the ovaries and sewing them back in. Starting from one week after the surgery, all rats accepted either EGCG or the saline vehicle every day for 8 weeks.

### 2.2 Behavioral test

Anxiety‐related activities were analyzed in open field test (OFT) and elevated plus maze (EPM) test. All behavioral analyses were carried out between 09:00 and 14:00, recorded using a visual tracking system (EthoVision XT 8.5 for video tracking) and monitored by a trained observer blinded to the precedent procedures.

### 2.3 Open field test (OFT)

A 5‐min OFT was carried out to investigate exploratory behavior and anxiety‐related behavior in rodents. All rats were subjected to OFT setup according to previous literature (Carrey, McFadyen, & Brown, 2000). After 24‐hr interval since the last dose of drugs was administered, animals were released onto the floor of the open field squares (100 cm × 100 cm × 40 cm) which were electronically divided into 9 equal squares, surrounded by base and walls made of nonreflecting gray material. The central square was defined as the ‘‘center’’ region. The initial placement of each rat was in a corner square, facing the wall, and the observation lasted for 5 min. The total travelled distance was associated with activity; and the frequency of entries into the center region was associated with emotionality; these data were automatically recorded. As a result of OFT, the relief of anxiety‐like behavior was proved by more time spent in the center region (Hiroi & Neumaier, 2011). The OFT apparatus was carefully cleaned according to laboratory protocols after each session in all behavioral experiments.

### 2.4 Elevated plus maze (EPM) test

All groups of female rats were subjected to EPM which is widely used to test anxiety‐like behavior and responses (Pellow, Chopin, File, & Briley, 1985). The maze was raised 80 cm above the floor, made of dark gray Plexiglas walls, and includes four arms (50 cm in length and 10 cm in width); two of the arms were closed arms with ledges of 40 cm in height, and two arms were open arms with ledges of 0.5 cm in height. A central square connecting the arms measured 10 cm × 10 cm. Individual animal was placed in the central square facing an open arm at the start. Then, the observation lasted for 5 min using a video camera set above the cage and was analyzed with tracking software. The time spent in the open arms and the frequency of entries into the center of the maze were noted. The EPM apparatus was carefully wiped clean after each test. It was presumed that the animals were more affected by anxiety or similar emotions if they spent more time in the closed arms.

### 2.5 Preparation of specimens

After all behavior tests, the rats were anesthetized by overdose of chloral hydrate and sacrificed by exsanguination. Before being anesthetized, fecal samples were collected in tubes. Blood samples for estradiol concentration assay were collected by puncturing the abdominal aorta before death and bleeding into tubes. Then, serum was extracted with centrifuge (1,000 *g* for 10 min) and stored at −80℃.

### 2.6 Determination of estradiol concentration

The plasma level of estradiol was measured by ELISA assay performed at automated instrument made by Beijing Science and Technology Co., Ltd. (ARB12673). All measurements were conducted and controlled up to the standard manual.

### 2.7 PCR and sequencing

Fragments in hypervariable regions of 16S V4 rDNA were amplified with specific primer set (515F: 5ʹ‐GTGCCAGCMGCCGCGGTAA‐3ʹ, 806R: 5ʹ‐GGACTACHVGGGTWTCTAAT‐3ʹ). The conditions for PCR steps were stated as the following: incubation at 98°C for 3 min; 30 cycles at 98°C for 45 s, 55°C for 45 s, and 72°C for 45 s; 72°C for 7 min; and holding at 4°C. The products of PCR were purified with Ampure XP beads (AGENCOURT) to remove the unspecific products. The sequencing of the product was performed by Illumina HiSeq 2500 PE250 (Illumina) following the instrumental manual. The library was established based on the MiSeq (Illumina) platform.

### 2.8 Statistical analysis

One‐way ANOVA with LSD was used for statistical analysis among groups to assess the homogeneity of variance, and the rank test using the Kruskal–Wallis method was used to analyze missing variance data. Calculations were done by the SPSS version 11.5 software (SPSS Inc.). *p* < .05 indicated statistically significant.

## 3 RESULTS

### 3.1 Effect of EGCG on the level of serum estradiol

**Figure 1:**
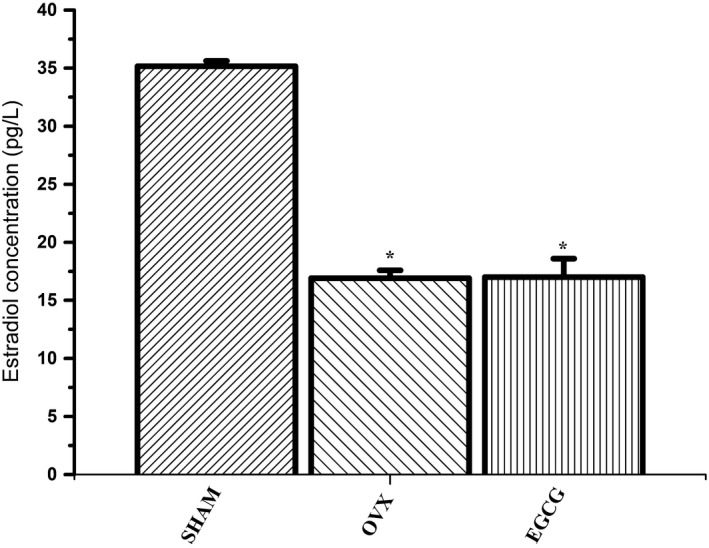
The effect of EGCG on serum estradiol level as the result of 8‐week treatment. *p < .01, compared with SHAM group

Figure 1 showed the levels of serum estradiol in different groups after 8‐week EGCG administration. The levels of serum estradiol in OVX group were lower compared with SHAM group (*F* = 267.058, *p* < .01). However, the level of serum estradiol for EGCG group rats showed no significant difference compared with OVX group.

### 3.2 The effect of EGCG on anxiety‐like behaviors of OVX rats in EPM and OFT

**Figure 2:**
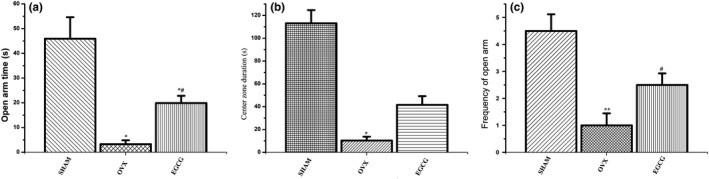
Anxiety‐like behaviors in EPM. 2A shows open arm time duration(s) for the three groups. EGCG treatment ameliorated the anxiety‐like symptom compared with OVX group (one‐way ANOVA with LSD). 2B shows EGCG treatment decreased center zone stay of OVX animals compared with SHAM group and EGCG group (one‐way ANOVA with LSD). 2C shows that the frequency of entries in open arms in EPM apparatus was significantly increased in EGCG group compared with OVX group (Rank test). *p < .05, **p < .01, compared with SHAM group; #*p* < .05, compared with OVX group

As observed in Figure 2a, the time spent by OVX rats in open arms was significantly reduced compared with SHAM group and EGCG group rats (*F* = 16.043, *p* < .01, Table 1). In addition, center zone duration in EPM apparatus was less for OVX rats compared with SHAM group rats and EGCG group rats (*F* = 41.066, *p* < .01, Figure 2b and Table 1). Moreover, frequency of open arm duration was less for OVX group rats compared with SHAM group rats (*F* = 13.605, *p* < .01, Figure 2c and Table 1). Therefore, OVX rats showed increase in anxiety‐like behavior. EGCG administration attenuated the anxiety‐like alterations in OVX rats.

**Table 1:** Total EPM: open arm time, center zone duration, and frequency of open arm of three groups

Group	*N*	EPM:open arm time(s)	Center zone duration(s)	Frequency of open arm
SHAM	6	45.93 ± 21.30	113.13 ± 28.29	4.50 ± 1.52
OVX	6	3.27 ± 3.82**	10.37 ± 8.12**	1.00 ± 1.09*
EGCG	6	19.90 ± 7.14#,*	41.60 ± 18.71**,#	2.00 ± 0.89

* *p* < .05, **p < .01 compared with SHAM group; #*p* < .05, compared with OVX group. Data are expressed as the means ± *SD* (*n* = 6 in all group).

**Figure 3:**
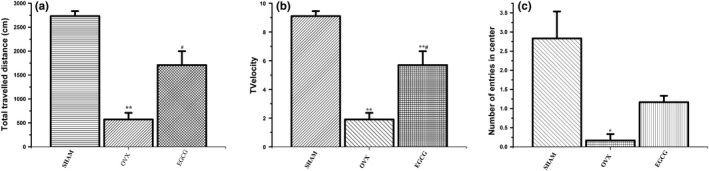
The effects of EGCG on the observations during OFT. 3A showed that the total travelled distance in EGCG group increased compared with OVX (*p* < .05, one‐way ANOVA with LSD). 3B showed that velocity of OVX group rats decreased compared with SHAM group (*p* < .01). 3C showed that frequency in center zone was less for OVX group rats compared with SHAM (*p* < .01, one‐way ANOVA with LSD) and EGCG group (*p* < .05, one‐way ANOVA with LSD). *p < .05, **p < .01, compared with SHAM group; #*p* < .05, compared with OVX group

During OFT, the total travelled distance and the number of entries for OVX group were significantly reduced compared with SHAM group, indicating an increase of anxiety‐like behavior in OVX group (*F* = 30.939, *p* < .05, Table 2 and Figure 3). Meanwhile, both the two measurements for EGCG group were higher compared with OVX group indicating the antianxiety effect of EGCG (*F* = 30.939, H = 13.107, respectively; *p* < .01). Velocity of OVX group rats was less compared with SHAM group rats, but EGCG group showed no difference with respect to velocity compared with OVX group (*F* = 30.939, *p* < .01).

**Table 2:** Total travelled distance, velocity, and number of entries in center of three groups

Group	Total travelled distance(cm)	Velocity	Number of entries in center
SHAM	2,732.27 ± 253.97	9.11 ± 0.85	2.83 ± 1.72
OVX	572.21 ± 338.74*	1.91 ± 1.13*	0.16 ± 0.41**
EGCG	1709.19 ± 707.23*,#	5.70 ± 2.36	1.17 ± 0.41#

* *p* < .05, **p < .01 compared with SHAM group; #*p* < .05, compared with OVX group. Data are expressed as the means ± *SD* (*n* = 6 in all group).

### 3.3 Operational taxonomic units

**Figure 4:**
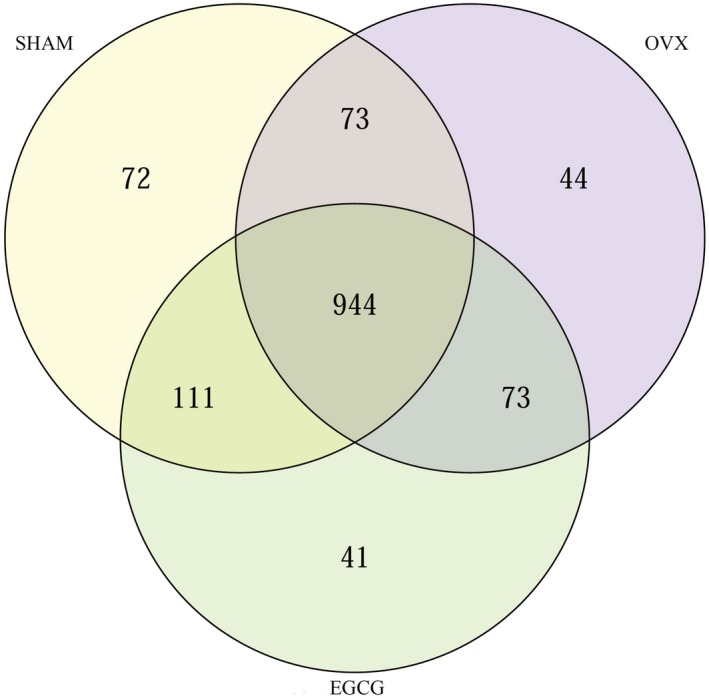
The distribution of OTUs for the three groups after 8‐week treatment of EGCG

Referring to sequence similarity (>97%), tags were clustered into multiple operational taxonomic units (OTUs) by software USEARCH (v7.0.1090). The abundance of each sample in each OTU was evaluated. The abundance of OTU preliminarily showed the species richness in the sample. The total richness of OTU generated from 16 samples was 1,358. The total of 1,200, 1,134, and 1,169 OTUs were found in the SHAM, OVX, and EGCG groups, respectively. The three groups shared 944 (69.51%) OTUs (Figure 4).

### 3.4 Alpha diversity analysis

Alpha diversity is an analysis of species diversity in a single sample, including observed species, with the indices of Sobs, Chao, ACE, Shannon, and Simpson. The larger the first four indices, or the smaller the last index, indicating the more abundance of species in the sample. Sobs, Chao, and ACE reflect the species richness of samples. Shannon and Simpson reflect species diversity and are influenced by species richness and species evenness. In this study, the OVX group had lower values of richness index (Sobs: 736, Chao: 848, ACE: 845, Shannon: 4.42) compared with SHAM group (Sobs: 798, Chao: 894, ACE: 890, Shannon: 5), while EGCG group had higher values in richness index (Sobs: 757, Chao: 869, ACE: 858, Shannon: 4.93) compared with OVX group. Meanwhile, the OVX group had a higher species evenness index (Simpson: 0.044) compared with SHAM group (Simpson: 0.018) and EGCG group had a lower species evenness index (Simpson: 0.021) compared with OVX group. The differences revealed that the samples of EGCG group showed enhanced biodiversity than those of OVX group (Table 3).

**Table 3:** Estimation of diversity within fecal samples from the three groups

Group	sobs	Chao	Ace	Shannon	Simpson
SHAM	798	894	890	5	0.018
OVX	736	848	845	4.42	0.044
EGCG	757	869	858	4.93	0.021

### 3.5 β‐diversity analysis

**Figure 5:**
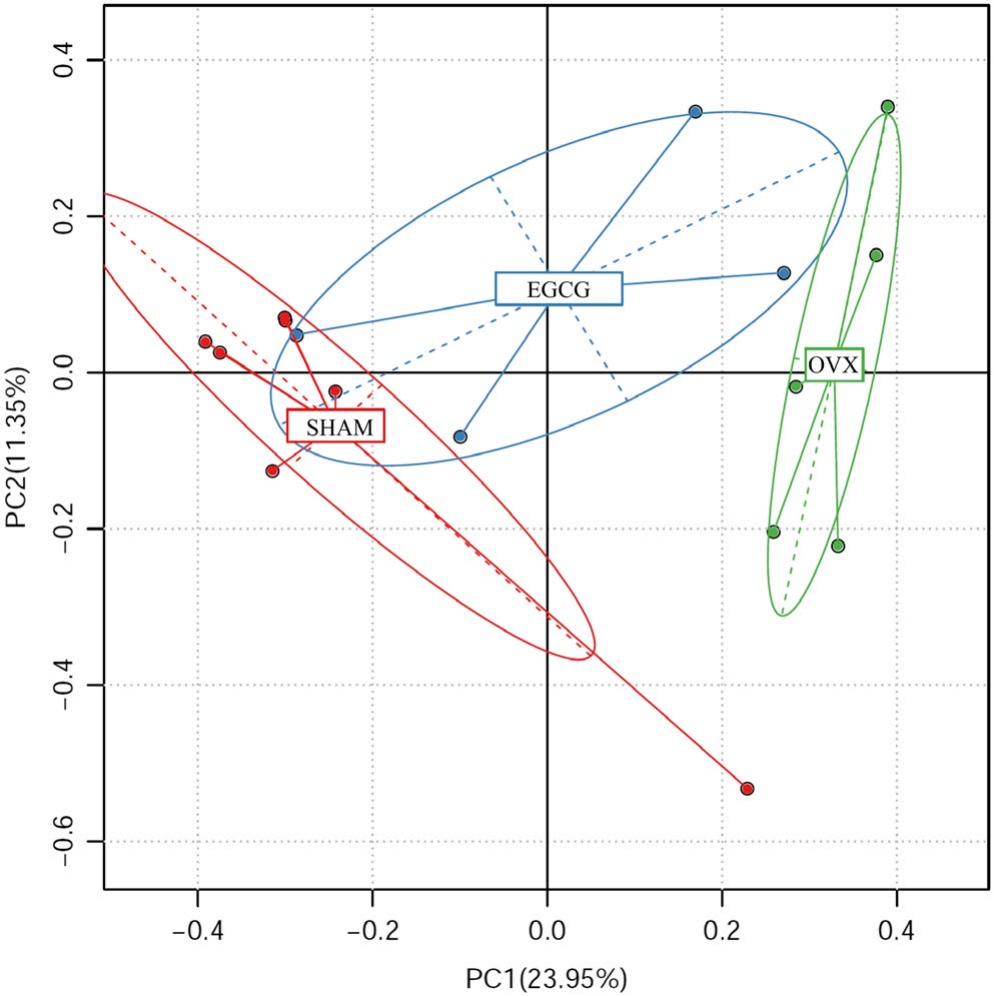
PCA plot of microbial communities from 16 fecal samples. PCA denotes principal component analysis. The percentages of variation in terms of principal components were showed on the axes. Red denotes SHAM group, green denotes OVX group, and blue denotes EGCG group

The β‐diversity of each group was assessed with principal component analysis (PCA) referring to the differentiated OTUs for SHAM, OVX, and EGCG groups. By analyzing the OTU (97% similarity) composition of different samples, the differences and distances between groups can be identified. PCA applied variance decomposition for comparing multiple sets of data in a 2D coordinate system. The two axes represented two values of variance maximum. The abscissa represented the first principal component, and the percentage in brackets represents the contribution of the first principal component to the difference between samples; the ordinate represented the second principal component, and the percentage in brackets represented the contribution of the second principal component to the difference between samples. The points in the picture represented each sample separately. Different colors represented samples belonging to different groups. Figure 5 showed that most samples in SHAM group and EGCG group were distributed close to each other, while OVX group was more isolated from these two, indicating that the overall composition of fecal microbiota in SHAM and EGCG groups shared more similarity compared with OVX group. The percentages of PC1 and PC2 were 23.95% and 11.35%, respectively, indicating variation.

### 3.6 Several phylum and genus differed between OVX and EGCG

At the phylum level, EGCG group had higher relative abundance levels of Actinobacteria, Verrucomicrobia, and Firmicutes compared with OVX group (Table 4). At the genus level, it was also found that the abundance levels of several genera in EGCG group including Bacteroidetes were increased compared with OVX group (Table 5).

**Table 4:** Differential gut microbiota at the phylum level

Taxa	SHAM	OVX	EGCG	*p* value
Actinobacteria	0.16 ± 0.08	0.06 ± 0.02	0.13 ± 0.04	.038
Firmicutes	50.35 ± 9.5	38.32 ± 6.99	57.04 ± 6.95	.017
Verrucomicrobia	0.31 ± 0.533	5.85 ± 3.81	0.45 ± 0.68	.0076

Note

Data are expressed as the means ± *SD* (*n* = 6 in SHAM group, *n* = 6 in OVX group, *n* = 4 in EGCG group).

**Table 5:** Differential gut microbiota at the genus level

Taxa	SHAM	OVX	EGCG	*p* value
Akkermansia	0.31 ± 0.53	5.85 ± 3.81	0.45 ± 0.68	.007
Anaerotruncus	0.0013 ± 0.0018	0.0047 ± 0.0034	0.007 ± 0.0035	.02
Anaerovibrio	0.09 ± 0.21	0.004 ± 0.008	0.006 ± 0.01	.03
Bacteroides	5.72 ± 3.83	1.87 ± 0.49	2.01 ± 0.48	.009
Butyricimonas	0.011 ± 0.006	0.006 ± 0.004	0.003 ± 0.001	.03
Escherichia	0.006 ± 0.007	0.124 ± 0.139	0.007 ± 0.007	.035
Phascolarctobacterium	0.663 ± 1.654	0.95 ± 0.37	0.512 ± 0.413	.049
Prevotella	11.31 ± 11.65	26.76 ± 12.88	9.8 ± 3.87	.02
Rothia	0.036 ± 0.027	0.007 ± 0.005	0.045 ± 0.019	.02
Veillonella	0.0136 ± 0.012	0.05 ± 0.03	0.09 ± 0.08	.01
vadinCA11	0.006 ± 0.009	0.0004 ± 0.0009	0.013 ± 0.02	.047

Note

Data are expressed as the means ± *SD* (*n* = 6 in SHAM group, *n* = 6 in OVX group *n* = 4 in EGCG group).

## 4 DISCUSSION

In this study, after ovariectomy, serum estradiol level for SHAM group was increased compared with both the OVX and EGCG groups, which confirmed the findings in precedent literature (Zhang et al., 2017). Behavior tests showed that treatment of ovariectomized rats with EGCG greatly enhanced their exploratory behavior and activity during OFT and EMP tests. This suggested that EGCG treatment had an antianxiety‐like effect. Furthermore, our current findings showed that, compared with SHAM group, the species richness decreased and the species evenness increased significantly in OVX rats, and EGCG treatment enhanced the richness but reduced the evenness, consequently increased diversity of the fecal microbiota.

More than 80% of women would suffer various degrees of psychological and/or physical abnormalities during menopause (Gracia & Freeman, 2018). The persistent absence of ovarian hormones following ovariectomy in rats was proposed to be the animal model for early postmenopause stage (Bosee & Di Paolo, 1995). Anxiety is a common phenomenon in menopausal female of both human and animals (Dalal & Agarwal, 2015; Dornellas et al., 2018). In this study, the ovariectomized group rats exhibited anxiety‐like behavior as observed through OFT and EPM. This corroborated the findings of Fedotova et al. that the anxiety‐like state is enhanced in OVX rats compared with normal rats (Fedotova, Pivina, & Sushko, 2017). Meanwhile, OVX rats which were treated with EGCG showed a decreased anxiety‐like state compared with untreated OVX rats. The results indicated that EGCG was able to decrease anxiety‐like activities which is in agreement with many previous studies (Park et al., 2010; Vignes et al., 2006).

Increasing number of studies have reported that anxiety and depression may be influenced by microbiota–gut–brain axis (Rieder et al., 2017). The underlying associations between microbiota and psychopathological conditions had previously been recognized, for example, microbiota alteration was involved in hypersensitivity of the hypothalamus–pituitary–adrenal (HPA) axis in response to stressors (Sudo et al., 2004), and psychological stress could cause microbial translocation resulting increased gut inflammation (Rieder et al., 2017). Therefore, the millions of bacteria in the gut may substantially influence on mental disorders, including anxiety and depression.

However, few studies have focused on how ovariectomy could be related to the alterations of gastrointestinal microbiota. Zhang et al and Wang et al verified that intestinal microbiota was altered in OVX rats. The abundances of two major phyla of gut microbiota populations were altered: Populations of Bacteroidetes were decreased, and Firmicutes populations had the trend to increase (Wang et al., 2016; Zhang et al., 2017). Furthermore, another study found that the ratio of populations of Firmicutes to Bacteroidetes in OVX mice was increased (Jin et al., 2018). The present study also found the OUT in OVX rats was different from SHAM rats. At the phylum level, Actinobacteria, Verrucomicrobia, and Firmicutes showed a higher population in SHAM and EGCG groups compared with OVX group. At the genus level, 11 genera in EGCG group or SHAM group were different from that in OVX group, including Bacteroidetes. Our results indicated that EGCG could modulate the intestinal microbiota in OVX rats.

EGCG exhibits a wide range of physiological functions, including the anticancer, anti‐hyperlipidemia, anti‐inflammation, and anti‐hypertension effects (Legeay, Rodier, Fillon, Faure, & Clere, 2015). EGCG also exhibits the effect of preventing bone loss in ovariectomized rats. Recent studies have found that EGCG also exhibits antianxiety effects (Park et al., 2010). Moreover, Most, Penders, Lucchesi, Goossens, & Blaak (2017) found that supplementation of EGCG + RES for 12 weeks could affect the gut microbiota composition in men but not in women. It indicated that EGCG may affect the gut microbiota composition in menopausal women who showed lower estradiol concentration. Our study found that treatment with EGCG for 8 weeks could affect the gut microbiota composition, and at the same time, EGCG also affected the anxiety‐like behavior in OVX rats which is a famous model for menopausal women. Thus, these findings illuminated the effects of EGCG on neuropsychic diseases, and it revealed that EGCG could be a drug of great potential for menopausal women in the future. However, more evidence was still required to verify that EGCG exhibits antianxiety effects by modulating intestinal microbiota.

## 5 CONCLUSIONS

The present study is the first to reveal that dietary EGCG not only affects the diversity of gut microbiota but also improves anxiety symptoms in ovariectomized rats. At the phylum level, Actinobacteria, Verrucomicrobia, and Firmicutes showed higher abundance in SHAM and EGCG groups compared with OVX group. At the genus level, 11 genera in EGCG group or SHAM group were different from those in OVX group, including Bacteroidetes. Based on the findings, it was further assumed that the diversity of gut microbiota and anxiety in OVX rats are strongly related supported by the simultaneous effects of EGCG. Although the deeper mechanistic links need further exploration, such as the explanation of relevant molecular signal pathway, it still would be expected that EGCG had a great potential in ameliorating the symptoms of anxiety in postmenopausal women.

## CONFLICT OF INTEREST

The authors declare that they have no conflict of interest.

## ETHICAL APPROVAL

The study was carried out at Department of Physiology, College of Basic Medical Sciences, Jilin University. Approval from the Ethical Committee of the College of Basic Medical Sciences, Jilin University, was obtained (Decision Date: 05.11.2018), and all animals received care in compliance with the “Principles of Laboratory Animal Care.”
